# Pertinent issues in pretransplant recipient workup

**DOI:** 10.4103/0970-1591.33725

**Published:** 2007

**Authors:** Pranjal Modi

**Affiliations:** Institute of Kidney Diseases and Research Centre and Institute of Transplantation Sciences, Civil Hospital Campus, Ahmedabad - 380 016, India

**Keywords:** Atherosclerosis, dialysis, kidney, nephrectomy, sexual dysfunction, transplantation, urinary bladder

## Abstract

Renal transplantation is recognized as the treatment of choice in most patients with end-stage renal disease. The evaluation of the candidate for kidney transplantation has been the recent subject of clinical practice guidelines published by the European Renal Association- European Dialysis Transplant Association and the American Society of Transplantation. The purpose of this article is to review the current literature for urological evaluation and treatment of patients prior to renal transplantation. In India, urologists are involved in evaluating not only the genitourinary problems but also vascular access and, vascular anatomy and pathology especially related to major pelvic vessels. Hence, evaluation of the transplant recipient should include assessment of vascular access for hemodialysis, access for peritoneal dialysis, assessment of pelvic vessels to which renal allograft vessels need to be anastomosed and genitourinary system. In addition, review of the serological tests for infective viral diseases like hepatitis and human immunodeficiency viruses should always be done before starting clinical evaluation. A note of the evaluation performed by other specialists like nephrologist, cardiologist, endocrinologist, pulmonologist, anesthetist etc. should always be reviewed.

Renal transplantation is recognized as the treatment of choice in most patients with end-stage renal disease (ESRD). Despite an increased risk of death in the early post-transplant period, transplantation improves long-term survival and quality of life compared with dialysis.[1,2]

There are relatively few absolute contraindications to kidney transplantation. It is contraindicated in the context of active infection, malignancy, substance abuse or non-adherence to therapy or in cases where comorbidities are expected to limit life expectancy and the ability to benefit from kidney transplantation significantly.

Transplant candidates are referred either by their nephrologists or on occasion self-referred. Family members are encouraged to accompany the candidate to the transplant evaluation. Transplant candidates must understand that transplantation entails risks and potential complications, which may result in extended transplantation hospitalization, additional unscheduled clinical visits, blood tests and procedures and repeat hospitalization and lifelong immumosuppressants. Recipients who live a long distance from the transplant center are asked to plan to stay locally for the first month following transplantation.

The evaluation of the candidate for kidney transplantation has been the recent subject of clinical practice guidelines published by both the European Renal Association-European Dialysis Transplant Association and the American Society of Transplantation.[3,4] These guidelines are quite comprehensive and are valuable references for transplant centers. At many centers in India, urologists play a dominant role in renal transplantation. The purpose of this article is to review the current literature for urological evaluation and treatment of patients prior to renal transplantation.

In India, urologists are involved in evaluating not only the genitourinary problems but also vascular access and, vascular anatomy and pathology especially related to major pelvic vessels. Hence, evaluation of the transplant recipient should include assessment of vascular access for hemodialysis, access for peritoneal dialysis, assessment of pelvic vessels to which renal allograft vessels need to be anastomosed and genitourinary system. In addition, review of the serological tests for infective viral diseases like hepatitis and human immunodeficiency viruses should always be done before starting clinical evaluation. A note of the evaluation performed by other specialists like nephrologist, cardiologist, endocrinologist, pulmonologist, anesthetist etc. should always be reviewed.

## EVALUATION OF VASCULAR ACCESS

The native arteriovenous fistula (AVF) at the wrist is generally accepted as the vascular access of choice in hemodialysis (HD) patients, due to its low complications and high patency rates.[5] Recent clinical practice guidelines recommend the creation of vascular access (native fistula or synthetic graft) before the start of chronic HD therapy to prevent the need for complication-prone dialysis catheters.[6–8] The placement and adequate maturation of AVF before the initiation of HD therapy requires timely patient education and counseling, selection of the preferred renal replacement modality, selection of an access type and location and creation of the access at least several weeks to months in advance of its expected need.

### Preoperative assessment of the vessels

Nondominant forearm should be used for creation of arteriovenous fistula. Veins in the selected arm for construction of AVF should not have many venipunctures, intravenous catheter or thrombosis. In obese patients or in conditions when vessel cannot be assessed clinically, duplex sonography is a useful noninvasive method for evaluating the morphological and functional characteristics of vessels prior to AVF construction. The immediate success and flow rate of a newly constructed AVF is mainly dependent on arterial inflow and venous outflow. In complex cases, particularly in patients with a history of previous failed fistulas or prior vein cannulation, vein mapping using duplex sonography is an additional valuable tool. Also, in patients who previously had chronic cannulation of the subclavian or jugular veins, the central veins should be evaluated by duplex ultrasonography or venography to exclude any underlying stenosis or occlusion.[9] Thrombosis of fistula is more likely in patients with systemic lupus erythematosus and having procoagulable state like presence of anticardiolipin antibodies, deficiency of protein C, deficiency of protein S and abnormality of antithrombin III status. Postoperative anticoagulants should be used routinely to avoid recurrent thrombosis of vascular access in this group of patients.

The elbow AVF is a reliable means of establishing vascular access for HD, if a primary AVF at the wrist is technically not possible or as a secondary procedure. The transposition of the autologous basilic vein to the brachial artery provides suitable vascular access in the absence of a superficial vein, owing to the fact that this vein is not usually damaged by cannulation. The procedure is usually accompanied by ‘superficialization’ of the vein for easier cannulation.

### Monitoring of arteriovenous fistula

Regular inspection and examination of the fistula is advisable every month to detect the development and progression of stenosis in time and to prevent any eventual thrombosis, so that one is not forced to surgically correct an established thrombosis. The final trigger causing thrombosis is the critical reduction of arterial blood flow below 200 mL/min. Several procedures help to recognize critically low blood flow rates: auscultation (high frequency bruits at the site of stenosis), hand elevation (collapse of the poststenotic venous segment and persisting congestion of the prestenotic segment), prolonged bleeding after removal of the needle and elevated venous inflow pressure during dialysis treatment. Duplex sonography offers the advantage of morphologic and functional information.

## PATIENT WITH PERITONEAL DIALYSIS CATHETER

The success of chronic peritoneal dialysis (PD) depends to a large extent on the success of chronic peritoneal dialysis catheter.[10] Infection and mechanical complications continue to be a major problem in the management of patients on peritoneal dialysis. A number of different methods of insertion of the peritoneal dialysis catheter have been proposed in an attempt to reduce these complications. These techniques include surgical placement by open access to the peritoneum, blind percutaneous placement using a Tenckhoff trocar, blind percutaneous placement using a guide-wire (Seldinger technique), minitrocar placement using peritoneoscopy (Y-TEC) or laparoscopy, the Moncrief-Popovich technique (the segment of the catheter that is usually brought out through the skin is buried subcutaneously and the entire wound is closed for four to six weeks before the distal segment of the catheter is brought out through the skin via a small incision 2cm distal to the subcutaneous cuff) and the presternal catheter (a modified Swan-neck Missouri coil catheter which is composed of two silicone rubber tubes that are connected at the time of insertion). There is no technique of insertion of a peritoneal dialysis catheter that has consistently proven to be superior in the prevention of peritonitis.[11] Exit site for catheter should be away from the proposed incision for kidney transplantation. Recurrent or severe infection are the major causes of Tenckhoff catheter removal. It is the responsibility of a transplant surgeon to review exit site, tunnel or peritoneal infections and treat them with appropriate antimicrobial and antifungal agents and, removal of Tenckhoff catheter. Proper exit site care is of paramount importance to reduce Tenckhoff catheter-related infections and subsequent catheter losses.[12]

Omental excision is performed to avoid mechanical obstruction to flow of dialysing fluid through the catheter in the peritoneum during open excess placement. When one chooses to keep catheter percutaneously or laparoscopically omental excision is not performed. Exit site of catheter should not be at the fat mass causing folds and it should be convenient to patient to perform dialysis and ambulation. The common site is in the pararectal region just above the level of umbilicus. However, in obese patients and in children exit site could be in the epigastrium or hypochondrium.

## EVALUATION OF PELVIC VESSELS

Atherosclerosis frequently affects the iliac arteries in patients with end stage renal failure. Impaired endothelial function in chronic renal failure promotes the progression of structural lesions of the arterial wall.[13] The initial arterial assessment includes femoral artery palpation, X-ray film of the abdomen and color Doppler ultrasound. Additional studies like computerized tomography, magnetic resonance angiography and/or arteriography in select cases.[14] Patients with diffuse, symptomatic aortoiliac atherosclerosis or symptomatic peripheral arterial atherosclerosis of the legs should be considered candidates for pre-transplant vascular bypass or excluded from transplantation. Arterial restoration during renal transplantation should now be less frequent due to better preoperative screening and the prevention of arteriosclerosis in patients on renal transplantation waiting lists but in some patients external and/or internal iliac atheroma may require an additional surgical vascular procedure during renal transplantation.[15] Aortoiliac lesions in a renal transplant candidate warrant consideration of staged or simultaneous arterial reconstruction.[16–21] However, one needs to consider the risk of prolonged surgery and renal artery thrombosis when aortobifemoral bypass is performed at the same time as renal transplantation. Thromboendarterectomy of the atherosclerotic vessels has a risk of stenosis or arterial thrombosis and orthotopic allograft placement can be a good choice for recipients with severe pelvic atherosclerosis.[22,23] Very severe pelvic vessel disease may be a significant cause of technical graft failure and may enhance the risk of limb amputation.[24]

## EVALUATION OF GENITOURINARY ORGANS

### Lower urinary tract

The basic urological assessment should include a detailed history, physical examination with special attention to the location of scars, abdominal catheters and abdominal stomas that may interfere with vascular surgery of renal transplantation or the connection to the urinary bladder, midstream urine analysis, urine or bladder wash culturing and abdominal and pelvic ultrasound in all patients. Urine volume voided in 24h should be measured which may be helpful to determine amount of fluid intake and, sometimes in post-transplant phase would patient has condition of rising serum creatinine value with good urine output from native kidney. We routinely perform X-ray KUB for two reasons: stone disease is endemic and often vessels are calcified in chronic kidney disease.[4,25] Depending on the presence or detection of urinary tract disease, additional tests may be required. For symptomatic patients who still make a reasonable quantity of urine, a voiding diary that documents urinary continence, voided volumes and times of voiding is helpful to determine the need for urodynamic evaluation. Standardized questionnaires can be used to document voiding symptoms and additional studies to evaluate the urinary tract are performed when indicated [Table 1, 2].[26]

**Table 1:** Excerpts from self-administered kidney transplant health questionnaire

Genitourinary	
Do you urinate every day?	Yes/No
Do you wet yourself?	Yes/No
Do you have to strain to empty your bladder?	Yes/No
Have you ever had to empty your bladder with a catheter?	Yes/No
Do you get up every night to empty your bladder?	Yes/No
Have you ever seen blood in your urine?	Yes/No
Have you ever had an infection in your urine?	Yes/No
Have you ever had kidney pain?	Yes/No
Have you ever had a kidney stone?	Yes/No

List all your operations and the approximate dates. List any cancer that you have had

**Table 2:** Indications for additional lower urinary tract studies in renal-transplant candidates

Studies	Indications
VCUG+/−urodynamics	Voiding dysfunction, bladder augmentation or substitution, history of pyelonephritis or reflux, inconclusive ultrasonography
Cystoscopy	Suspected lower urinary tract cancer or planned invasive prostate therapy
Urine or bladder wash cytology	Prior cylcophosphamide therapy or significant irritative voiding symptoms
Bladder biopsy	Suspected bladder fibrosis or cancer

Incidence of genitourinary abnormalities requiring specific therapy in patients with no urologic history is extremely low.[27,28] Hence, routine urologic assessment beyond a history and physical examination is not warranted.[29] Voiding cystourethrogram (VCUG) is not uniformly required as part of a pretransplantation evaluation of a potential adult recipient. The reason for a very low yield of VCUGs in adults is that the prevalence of total urologic abnormalities in the adult patient population with ESRD is less than 5%.[30] The need for a VCUG, cystoscopy or a retrograde pyelogram should be determined on an individual basis during the pretransplant surgical assessment. Even in children with end stage renal disease due to etiology other than urological disease pretransplantation voiding cystourethrography is not necessary.[31] In patients without a urologic history a good ultrasound is reliable in evaluating the kidneys and bladder. If the kidneys are uniformly smooth in outline without focal scars and the urinary bladder shows no evidence of thickening one should proceed with transplantation in these patients without preoperative VCUGs.

Any prospective pediatric kidney recipient with a urologic basis for end stage renal disease or with any history of urologic problems, should definitely have a VCUG performed pretransplantation. This group includes patients with a history of congenital obstructive uropathy, urinary tract infections, renal hypodysplasia and defunctionalized urinary bladders. It is important to note that most of these patients have already had at least one such study performed prior to transplantation.

About 6% of patients undergoing renal transplantation each year in the USA have ESRD secondary to a lower urinary tract abnormality.[32] Such abnormal bladder and its outlet must be modified to render kidney transplantation safe or the graft must be transplanted into some form of urinary diversion. Assessment of the suitability of native bladder for drainage of transplanted allograft is an important issue. Patients with urinary diversions whose native bladders remain *in situ* and those who continue to void per urethra but have oliguria or anuria, have bladders which have been defunctionlaized and are likely to have low bladder capacities. Low bladder capacity before surgery does not preclude safe transplantation and, after transplantation bladder capacity enlarges progressively.[33] Various methods of ‘bladder cycling’ to help establish the likelihood of normal bladder capacity and compliance have been described.[33,34] However, its value in pretransplant assessment remains unclear. In patients whose end stage renal disease is caused by either a congenital malformation (i.e. posterior urethral valve, spina bifida, prune belly, vesicoureteric reflux, bladder extrophy, VATER (vertebral/vascular anomalies, anal atresia, tracheo-esophageal fistula, esophagial atresia, renal anomalies/radial dyaplasia)) or by a functional disorder of the lower urinary tract, the abnormality must be corrected 10–12 weeks before transplantation, with pretransplant urodynamic assessment being the key investigation.[35,36]

Appropriate urinary drainage is required for successful transplantation, the need for urologic surgery before transplantation should be carefully assessed in patients with a dysfunctional bladder. Many patients can be managed with intermittent self-catheterization, but some patients may require bladder augmentation or urinary diversion before transplant. Morbidity and quality of life are superior with intermittent self-catheterization compared with surgical approaches.[37] Reconstructive bladder surgery like augmentation cystoplasty, continent reservoir, ileal conduit or orthotopic neobladder before transplantation is advocated for patients having small contracted bladder and/or problem of bladder outlet.[38–40] Most patients with augmentation cystoplasty require clean intermittent self-catheterization before and after transplantation.[41] The augmented bladders commonly become colonized with gut flora. The role of prophylactic antibiotics has not been studied in a controlled fashion. If the patient is asymptomatic and pyelonephritis does not ensue, bacteriuria is usually not treated. An exception is colonization with urea-splitting organisms such as *Proteus mirabilis* because this may lead to the formation of struvite stones.[26] Others, however, believe in regular drainage of an augmented bladder along with low-dose antibiotic prophylaxis to prevent symptomatic urine infections.[38] Functional augmentation is preferable to dry augmentation because it permits continence and bladder compliance to be documented before transplantation.[42] In anuric population the ‘recycling bladder regimen’ in the interval after reconstructive bladder surgery and before transplantation is suggested to prevent the decrease of capacity and compliance secondary to the defunctionalization of the reservoir.[43]

Electrolyte and metabolic abnormalities can occur in the augmented urinary bladder.[42] Acidosis should be treated because of its contribution to metabolic bone disease. The mucous problem common to intestinal procedures can be dealt with by daily bladder irrigations. The megaloblastic anemia associated with the use of the distal ileum can be treated with vitamin B12. Bladder and upper-tract stones occur in 8–52% of patients with bladder augmentation.[44,45] Struvite stones in the kidney and bladder are more common after use of bowel in urinary tract. Prior to transplantation the patient should be free of both stone and infection. Dilated ureter can be used for augmentation cystoplasty to avoid problems related to absorption of urinary metabolites and electrolyte imbalance with use of bowel.[46]

High-grade vesicoureteric reflux (VUR) that is left untreated post transplantation is associated with an increased risk of urinary tract infection, even if urinary tract infection was not a problem before transplantation.[47] Surgical options for treatment—ureteric reimplantation or nephrectomy—have been associated with a reduced risk of infection post transplantation.[48,49] Endoscopic collagen injection has been used successfully to treat children with VUR (including during preparation for transplantation) and is associated with less morbidity than surgery.[50–52] Although no approach is specifically favored, the combination of megaureter and an associated non-functioning kidney may present a heightened risk for infectious complications post transplantation and nephrectomy may be preferred.

## GENITOURINARY MALIGNANCY

### Renal cancer

Patients on chronic dialysis may have acquired renal cystic disease (ARCD).[53] Patients with less than three years of dialysis have 10-20% incidence of ARCD, while 90% have ARCD after five years of dialysis.[54] Acquired renal cystic disease has been considered a factor predisposing to renal cell carcinoma.[53] Diagnostic criteria of ARCD are macroscopic cystic structures compromising at least 25% of the renal parenchyma or greater than three cysts per kidney.[55] Renal cell carcinoma in patients with ESRD grows at a rate of 0.5 to 1.0cm yearly.[56] Since most renal tumors grow slowly and approximately half of the patients with ESRD have ARCD after three years on dialysis, screening ultrasonography after Year 3 on dialysis and every other year thereafter is recommended.[57] Most RCCs are indolent until they are 3 cm and the risk of metastasis increases after tumors exceed 3 cm.

Patients with a history of symptomatic renal cell cancer have a recurrence rate of 30% following renal transplantation.[58] Of the patients with recurrent disease, 61% had been treated less than two years before transplantation and 33% between two and five years before transplantation.[58] Death due to recurrent disease may be as high as 80%.[4] The recurrence rate of incidentally discovered renal cell carcinoma is less than 1%.[4] Most patients with a past history of symptomatic renal cell carcinoma should wait at least two years from treatment to transplantation.[4] Large (>=5cm) or invasive renal cell cancers may require a five-year waiting period because of their higher risk of recurrence.[4] Small (<5cm), incidentally discovered renal cell cancers may not require any waiting period before transplantation.[4]

### Bladder cancer

Patients with preexisting bladder carcinoma have a recurrence rate of 18-26% following transplantation.[58,59] Most recurrences have been in patients who waited less than two years from treatment to transplantation.[58] Patients with a prior history of invasive bladder cancer should wait a minimum of two years from cancer treatment to renal transplantation. Patients with superficial lesions (pTa, uniform, Grade 1 disease) have a high risk of local recurrence (up to 60%) but a low risk of invasive or metastatic disease. These patients may not require any waiting period between treatment and transplantation,[4] but should undergo periodic surveillance with imaging of the upper urinary tract, urine cytology and cystoscopy both pre-and post-transplant. Carcinoma *in situ* is considered a high-grade lesion; such patients should undergo treatment and be disease-free for two years before renal transplantation.

There are few data to support cystoscopy as a routine screening procedure before transplantation. However, patients at high risk for cancer (analgesic nephropathy, cyclophosphamide use) should be considered for pretransplant cystoscopy.[4]

### Prostate cancer

Prostate cancer is common and affects 30% of men over the age of 50. One in 8-10 men will develop clinically significant prostate cancer. Most will be Gleason's Grade 3 disease with a doubling time of two to three years. The medium-risk population has a life expectancy of about 10 years if the cancer is untreated. Patients with preexisting prostate cancer have a recurrence rate of 18% following renal transplantation.[4,60] Those with localized disease (T_1_ and T_2_) have recurrence rates of 14-16% and those whose disease extends beyond the prostate capsule (T_3_+) have a recurrence rate of 36% and a mortality rate of 27%.[60] Of those with recurrent disease, 40% had been treated less than two years before transplantation.[60] Most patients with a past history of prostate cancer should wait at least two years between treatment and transplantation. Patients with advanced disease (outside the prostate capsules, T_3_+, T_4_, N+, M+) at presentation should not be offered transplantation because of the high risk of recurrence. Patients with low-risk prostate cancer may not require any waiting period.

### Testicular cancer

Patients with a history of testicular cancer have a recurrence rate of 3-12% following renal transplantation[58,61] with most (>75%) recurrences appearing within two years. Mortality due to recurrent disease ranges from 0-8%.[58,61] Most patients have waited more than five years before transplantation.[58,61] Patients with a history of testicular cancer should wait at least two years from treatment to transplantation.[4]

### Wilms’ tumor

Bilateral nephrectomy before transplantation is advocated for children with bilateral Wilms’ tumor or with the Denys-Drash syndrome to be certain of removing tissue with potential malignancy.[62] Survival, in general, is poorer for patients with bilateral Wilms’ tumor compared with unilateral disease.[63,64] The recurrence risk is greatest when transplantation is performed less than one year after completion of chemotherapy; thus, renal transplantation should be delayed until at least one year after completion of treatment,[62,64] although some advocate a delay of two years or more.[65]

## PRETRANSPLANT NATIVE KIDNEY NEPHRECTOMY

Pretransplant native kidney nephrectomy is indicated for recurrent or chromic pyelonephritis, structural abnormalities of the urinary tract predisposing the patient to infection, malignant or rennin-dependent hypertension, Goodpasture's disease and selected patients with polycystic kidneys.[66,67]

Hypertension in chronic renal failure patients is often difficult to control with multiple drugs in large dosages. With the administration of calcineurin inhibitors and steroids in the perioperative period problems of blood pressure control could be aggravated. Native kidney nephrectomy may reduce the need for large doses of antihypertensive drugs and eliminates its side-effects. Intraoperatively, intravenous drugs like sodium nitroprusside or nitroglycerine are sometimes required to avoid hypertensive crisis. Thorough hemostasis should be achieved during surgery to avoid excessive bleeding. Surgery in patients of chronic renal failure must be done with a team of urologist, nephrologists, intensivist and anesthetist having experience of dealing with per and perioperative fluid and electrolyte disturbances, management of acidosis, severe hypertension, cardiovascular instability and platelet dysfunction causing bleeding disorder. Appropriate antibiotics for either prophylaxis or therapy should be added to the perioperative period due to higher susceptibility to infection compared to non-renal-failure patients. Heparin should be avoided in the postoperative period, especially during dialysis to avoid bleeding from wound or drain site or collection of blood in kidney fossa.

Laparoscopic removal of one or both kidneys in patients with end stage renal disease is having less morbidity than open surgery.[68] Pure or hand-assisted laparoscopic bilateral simultaneous nephrectomy for ADPKD is safe and feasible.[69,70] Preoperative CT scan and/or magnetic resonance evidence of intracystic or pericystic hemorrhage should caution the surgeon to the dense perirenal adhesions at that location. Kidneys having volume over 3,500 mg should be removed by elective open surgery.[70]

## UROLITHIASIS

Urolithiasis is endemic in most parts of the country. Stone may be the cause of chronic renal failure or may be merely associated with medical renal disease. Combined ultrasonography of kidney and X-ray KUB region detect most of the cases of symptomatic and asymptomatic stone disease. Invasive investigations like retrograde pyelography may be carried out to detect small ureteric stone or when opacity in X-ray KUB region is suspected to be a ureteric or renal stone. It is prudent to review any chance of reversible renal function in case of obstructive uropathy. In the absence of any chance of recoverability of renal function nephrectomy with stone removal should be carried out prior to transplantation.

In patients with primary hyperoxaluria with end stage renal failure associated with multiple renal stones, bilateral native kidney nephrectomy followed by simultaneous or sequential kidney and liver transplantation should be performed. Systemic oxalosis causing severe cardiovascular dysfunction may preclude these patients from transplantation therapy.

## SEXUAL DYSFUNCTION

The prevalence of sexual dysfunction among patients of both sexes with chronic renal failure is high[71–73] which is primarily manifested as impotence and libido decrease in male patients and a decrease of sexual desire and lubrication defects in female patients. The origin is not well understood yet, although it may be attributed to the interaction of a series of factors:[71,73,74] uremic state, regressive issues conditioned by the chronic illness, inadequate physiological response to dialysis, decreased wellbeing, hormonal impairments, associated pathology (diabetes mellitus, vascular disease, uremic neuropathy, depression), medications, etc. Patients often regain potency after transplantation, although recent studies showed that 48-56% of renal transplant recipients continue to have erectile dysfunction.[72,75]

The incidence of vasculogenic impotence after renal transplantation with internal iliac artery is about 10%.[76] However, following second renal transplant with the other internal iliac artery the incidence increases between 25 and 65%.[77,78]

Treatment approach for each patient is dependent on the systematic evaluation of the functional and psychosocial problems presented and assessment of the cause of sexual dysfunction.[79] Sildenafil is a well tolerated and effective treatment for erectile dysfunction in men receiving maintenance hemodialysis.[80,81] Sildenafil is not cleared by hemodialysis but hemodialysis may remove the endogenous inhibitors of metabolism of sildenafil and thereby restore the pharmacokinetics closer to that observed in patients with normal renal function.[82]

In conclusion, the urologist has to play a major part in evaluating the patient having end stage renal disease. Urogenital abnormalities are more commonly found in children than in adults undergoing pretransplant evaluation. A systematic approach to evaluate the patient by detailed history, physical examinations, ultrasonography and other necessary investigations to reveal the genitourinary abnormalities and pelvic and lower limb vasculature should be applied. Vascular access for dialysis should be evaluated periodically by physical examination and Doppler ultrasound for early diagnosis of stenosis. Both retro- and trans-peritoneoscopic surgery are feasible and safe in patients with end stage renal disease. Surgery in chronic renal failure patients requires multidisciplinary approach and is teamwork. Sexual dysfunction should be evaluated in patients of both sexes due to its high prevalence in renal failure.
